# Implementation of an Anticoagulation Practice Guideline for COVID-19 via a Clinical Decision Support System in a Large Academic Health System and Its Evaluation: Observational Study

**DOI:** 10.2196/30743

**Published:** 2021-11-18

**Authors:** Surbhi Shah, Sean Switzer, Nathan D Shippee, Pamela Wogensen, Kathryn Kosednar, Emma Jones, Deborah L Pestka, Sameer Badlani, Mary Butler, Brittin Wagner, Katie White, Joshua Rhein, Bradley Benson, Mark Reding, Michael Usher, Genevieve B Melton, Christopher James Tignanelli

**Affiliations:** 1 University of Minnesota Minneapolis, MN United States; 2 Information Technology Fairview Health Services Minneapolis, MN United States; 3 Department of Surgery University of Minnesota Minneapolis, MN United States; 4 College of Pharmacy University of Minnesota Minneapolis, MN United States; 5 School of Public Health University of Minnesota Minneapolis, MN United States; 6 Department of Medicine University of Minnesota Minneapolis, MN United States

**Keywords:** COVID-19, anticoagulation, clinical practice guideline, evidence-based practice, clinical decision support, implementation science, RE-AIM

## Abstract

**Background:**

Studies evaluating strategies for the rapid development, implementation, and evaluation of clinical decision support (CDS) systems supporting guidelines for diseases with a poor knowledge base, such as COVID-19, are limited.

**Objective:**

We developed an anticoagulation clinical practice guideline (CPG) for COVID-19, which was delivered and scaled via CDS across a 12-hospital Midwest health care system. This study represents a preplanned 6-month postimplementation evaluation guided by the RE-AIM (Reach, Effectiveness, Adoption, Implementation, and Maintenance) framework.

**Methods:**

The implementation outcomes evaluated were reach, adoption, implementation, and maintenance. To evaluate effectiveness, the association of CPG adherence on hospital admission with clinical outcomes was assessed via multivariable logistic regression and nearest neighbor propensity score matching. A time-to-event analysis was conducted. Sensitivity analyses were also conducted to evaluate the competing risk of death prior to intensive care unit (ICU) admission. The models were risk adjusted to account for age, gender, race/ethnicity, non-English speaking status, area deprivation index, month of admission, remdesivir treatment, tocilizumab treatment, steroid treatment, BMI, Elixhauser comorbidity index, oxygen saturation/fraction of inspired oxygen ratio, systolic blood pressure, respiratory rate, treating hospital, and source of admission. A preplanned subgroup analysis was also conducted in patients who had laboratory values (D-dimer, C-reactive protein, creatinine, and absolute neutrophil to absolute lymphocyte ratio) present. The primary effectiveness endpoint was the need for ICU admission within 48 hours of hospital admission.

**Results:**

A total of 2503 patients were included in this study. CDS reach approached 95% during implementation. Adherence achieved a peak of 72% during implementation. Variation was noted in adoption across sites and nursing units. Adoption was the highest at hospitals that were specifically transformed to only provide care to patients with COVID-19 (COVID-19 cohorted hospitals; 74%-82%) and the lowest in academic settings (47%-55%). CPG delivery via the CDS system was associated with improved adherence (odds ratio [OR] 1.43, 95% CI 1.2-1.7; *P*<.001). Adherence with the anticoagulation CPG was associated with a significant reduction in the need for ICU admission within 48 hours (OR 0.39, 95% CI 0.30-0.51; *P*<.001) on multivariable logistic regression analysis. Similar findings were noted following 1:1 propensity score matching for patients who received adherent versus nonadherent care (21.5% vs 34.3% incidence of ICU admission within 48 hours; log-rank test *P*<.001).

**Conclusions:**

Our institutional experience demonstrated that adherence with the institutional CPG delivered via the CDS system resulted in improved clinical outcomes for patients with COVID-19. CDS systems are an effective means to rapidly scale a CPG across a heterogeneous health care system. Further research is needed to investigate factors associated with adherence at low and high adopting sites and nursing units.

## Introduction

COVID-19, caused by SARS-CoV-2, has infected millions of people worldwide. This disease has shown many unique attributes including a hypercoagulable profile [1-4]. COVID-19–associated coagulopathy results in widespread macrovascular and microvascular thrombosis that contributes to multisystem organ failure and thus contributes to significant mortality and morbidity [5]. Observational and recent randomized controlled studies involving COVID-19 and other viral pneumonias have suggested that routine anticoagulation is associated with improved clinical outcomes for hospitalized patients [6-10]. Considering this, our health care system developed a clinical practice guideline (CPG) delivered as a clinical decision support (CDS) system to facilitate guideline-driven anticoagulation for COVID-19 patients.

CDS technology solutions offer a mechanism that in support of the learning health system (LHS) facilitates long-term process and quality measure improvements [11,12]. CDS systems leverage electronic health records (EHRs) to deliver process-specific information to health care teams, aiding clinical decision-making. When designed well and implemented effectively, CDS systems have been shown to improve adherence with evidence-based practice and, in some cases, improve clinical outcomes [11-13]. Unfortunately, the best practices for successful implementation and scaling of CDS are still unknown [14]. Furthermore, very little is known about developing, implementing, and scaling CDS interventions during a pandemic with a rapidly changing evidence base and strained clinical resources across diverse sites.

**Figure 1:**
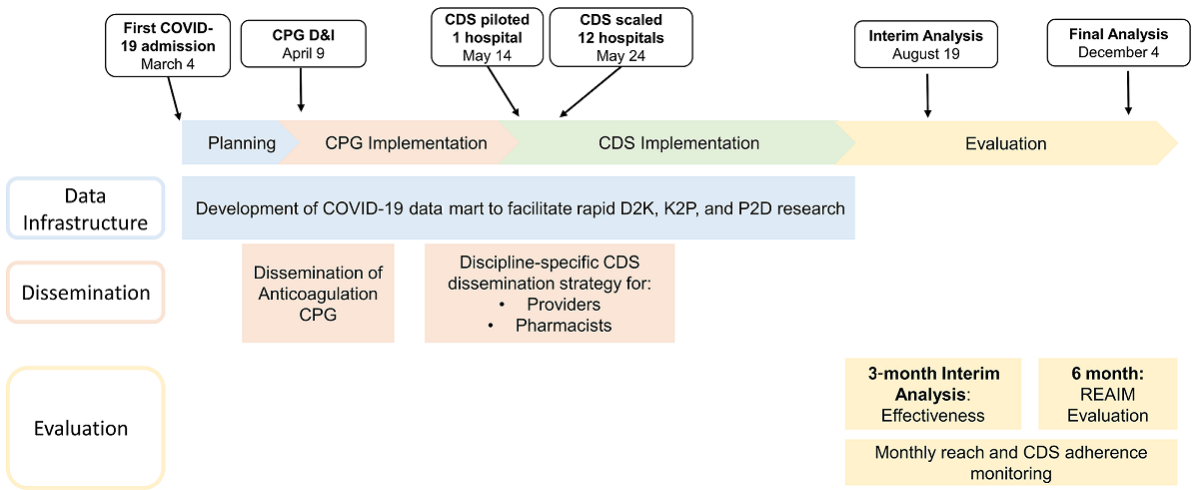
Overall development, dissemination, implementation, and evaluation strategy. CDS: clinical decision support; D&I: Dissemination and Implementation; CPG: clinical practice guideline; D2K: data to knowledge; K2P: knowledge to practice; P2D: practice to data; RE-AIM: Reach, Effectiveness, Adoption, Implementation, and Maintenance.

On April 9, 2020, our institution developed and disseminated a 3-tiered CPG for anticoagulation in COVID-19 in collaboration with national experts (Multimedia Appendix 1). Given the rapid evolution of evidence, this CPG has evolved over time to reflect the best practice based on the available evidence at the time [4,7]. To maximize the dissemination and reach of the CPG, a CDS solution was developed to deliver the CPG, including both passive and interruptive alerts, piloted at a single site on May 14, 2020, and was successfully scaled across a 12-hospital Midwest health care system on May 24, 2020. An interim reach, adoption, and effectiveness evaluation occurred 3 months following implementation on August 19, 2020 (Figure 1).

This study represents a preplanned 6-month implementation evaluation guided by the RE-AIM (Reach, Effectiveness, Adoption, Implementation, and Maintenance) framework [15] of the anticoagulation CPG CDS system for patients admitted with COVID-19 between March 4, 2020, and December 4, 2020, across a large 12-hospital Midwest health care system.

## Methods

### Context and Evidence Synthesis

A COVID-19 evidence-based medicine (EBM) team was created in March 2020 to rapidly review, catalogue, and publicly disseminate evidence related to proposed COVID-19 therapeutics including anticoagulation management [16]. Due to a lack of high-quality evidence from randomized controlled trials and a lack of expert guidelines, the EBM team developed a novel rubric to grade COVID-19 evidence [17]. Using this rubric, a multidisciplinary team, including COVID-19 physicians, LHS researchers, pharmacists, public health epidemiologists, and medical librarians, reviewed and graded the evidence to date for anticoagulation in COVID-19.

### CPG Development

Guided by the EBM team’s recommendations, system hematology service line leads (S Shah and MR) oversaw the development of a CPG in collaboration with national hematology experts (Multimedia Appendix 1). This CPG was initially disseminated beginning April 9, 2020. Significant controversy existed at the time regarding the appropriate anticoagulation strategy, with some studies suggesting no anticoagulation (*conservative treatment approach*) due to concerns for bleeding and disseminated intravascular coagulopathy, and some studies suggesting tissue plasminogen activator infusions (*aggressive treatment approach*) for patients with severe respiratory failure [8,18]. Ultimately, the CPG adhered to a “middle of the road” approach by instituting universal prophylactic weight-based anticoagulation for all patients. Similar to anticoagulation CPGs for other disease processes [19], we incorporated a risk stratification model, whereby the intensity of anticoagulation was increased to moderate intensity (0.5 mg/kg BID enoxaparin or low intensity heparin infusion in case of renal failure) for patients with a history of thrombosis, cancer, admission to the intensive care unit (ICU), or D-dimer >10 times the upper limit of normal. The CPG is a “living” framework, and has since undergone several iterations of modifications (upstratification of patients with a prior history of deep venous thrombosis or cancer, and ICU patients, and exclusion of pregnant patients) based on evolution of the evidence.

### CDS Development, Dissemination, and Implementation

The COVID-19 anticoagulation CPG CDS system was developed by the M Health Fairview clinical informatics development team (KK and PW) in collaboration with the Associate Chief Medical Informatics Office for CDS (S Switzer) in May 2020. Multimedia Appendix 2 displays a process map for the CDS system. In brief, the CDS solution includes the following (representative screenshots displayed in Multimedia Appendix 3 and Multimedia Appendix 4): a tiered anticoagulation order set, a passive “reminder” of the anticoagulation CPG for COVID-19 patients without anticoagulation orders displayed in the EHR admission and transfer navigator, 3 “triggers” that activate interruptive alerts, and various interactive and interruptive alerts.

The interruptive best practice advisories were essentially “safety checks” to surveil if patients were on venous thromboembolism (VTE) chemoprophylaxis on admission or if the criteria for VTE risk changed (eg, increase in D-dimer above the threshold or transfer to the ICU) and were only triggered for providers with ordering privileges. To ensure the best practice advisory would not trigger for patients who have recovered from COVID-19, an infection status of *Recovered COVID-19* was built into the EHR.

Development, dissemination, and implementation followed our system protocol SCALED (scaling acceptable CDS) for CDS user-centered design, pilot testing, scaling, and evaluation. Prior to pilot testing, the CDS underwent iterative user interface/user experience improvement during May 2020. The CDS was piloted on May 14, 2020, and scaled on May 24, 2020. To support adoption and usability, a discipline-specific CDS dissemination strategy was carried out in May 2020 (Figure 1). To ensure embeddedness of the CDS in provider and pharmacist workflow, dissemination overlapped with implementation for 1 month after the intervention went live or was “turned on.” The specific dissemination strategies are presented in Textbox 1.

**Dissemination strategies.**

**Providers**

- The clinical decision support (CDS) system was presented to intensivist, hospital medicine, and primary care practice groups via formal didactic methods.
- Our system utilized a daily workflow document for intensivists and hospital medicine providers caring for COVID-19 patients representing best practices, recent publications, and ongoing trials. The anticoagulation CDS was integrated into this workflow document and remained as a constant on this document throughout the implementation period.
- In the university setting, CDS was presented at grand rounds on divisional, departmental, and medical school platforms.

**Pharmacy**

- The CDS system was presented routinely at the System-wide Anticoagulation Committee.

**All**

- Our system utilized a COVID-19 intranet COVID-19 resource page. This CDS was placed within the system guidelines for management of COVID-19 patients.
- The CDS was also posted on the University of Minnesota evidence-based medicine COVID-19 website, on a public facing webpage for COVID-19 evidence-based practice geared toward clinicians.

### Data Extraction and Evaluation

Members of the study team (CJT, GBM, and MU) developed a COVID-19 data mart to facilitate near real-time evaluation of the CDS. Structured query language was used to automate daily export of COVID-19 EHR data into the data repository. A preprocessing pipeline was developed and implemented using Python 3.7.3. (CreateSpace) and Stata 16 (StataCorp) to generate a flat file for each patient, including patient anticoagulation risk stratification, tier of anticoagulation received, reach, adherence, clinical outcomes (in-hospital and out-of-hospital mortality, complications, ICU admission, and mechanical ventilation), comorbidities, home medications, inpatient medications received (eg, remdesivir, tocilizumab, and steroids), daily laboratory and vital data, and demographics. LogicStream Health (LogicStream Health Inc), an analysis platform for EHR content, was utilized to evaluate order set, passive, and interruptive alert utilization.

### CDS RE-AIM Evaluation

A preplanned 6-month implementation evaluation was conducted with guidance from the RE-AIM framework.

*Reach* was defined as the number of patients admitted each month, who received CDS (CDS reach) or appropriate anticoagulation (CPG reach) (numerator) over the number of patients admitted each month with COVID-19 (denominator). These 2 definitions of reach were used to facilitate internal performance monitoring. For example, the state where CPG reach is high but CDS reach is low represents integration of the CPG into normal workflow without the need for CDS.

*Adoption* was defined at the implementation site and nursing unit level as the number of patients admitted each month, who received guideline-concordant anticoagulation therapy (numerator) over the number of patients admitted each month with COVID-19 (denominator). It was not possible to define adoption accurately at the provider level as we were unable to assign a single provider responsible for a patient’s initial care. Patient’s receive orders from a variety of provider types, including house staff, advanced practice providers, or attending physicians either within the emergency department or the inpatient team, and thus we are unable to assign a single provider responsible for CPG adherence.

An *implementation* evaluation was conducted to investigate the effect of various CDS alert methods (passive and/or interruptive alerts) on anticoagulation CPG adherence. *Adherence* was defined at the patient level as receiving guideline-concordant care within 24 hours of admission. Additionally, CPG fidelity was evaluated for each VTE risk stratification (Multimedia Appendix 1).

To evaluate *maintenance*, following a wash-out period without any continued dissemination, we evaluated adherence during months 5 and 6 after implementation.

### Statistical Approach

To evaluate *effectiveness*, the association of CPG adherence on admission (at the patient level) with clinical outcomes was assessed via multiple methods.

First, multivariable logistic regression was used for binary dependent variables and negative binomial regression was used for continuous variables with a skewed distribution (hospital length of stay) using all 2406 patients who either received adherent care (n=1650) or did not receive adherent care (n=853). All models were risk adjusted using the confounding variables included below.

Second, 1:1 nearest neighbor propensity score matching was used to create cohorts of patients who received CPG-adherent care (exposure or treatment of interest). Univariate logistic regression was then used to compare the need for ICU admission within 48 hours (primary outcome) for patients who received (vs did not receive) CPG-adherent care on admission (exposure). Kaplan-Meier curves were also estimated via a time-to-event analysis (censored at 48 hours following hospital admission) and compared using the log-rank test. Propensity scores were estimated with logistic regression using the confounding variables listed below. Two evenly matched groups were formed with the common caliper set at 0.01. Following matching, there were 1342 patients included (671 patients in each propensity-matched cohort). Standardized difference was evaluated prior to and after propensity matching to ensure that the standardized difference was <0.1 in the propensity-matched cohort for each confounding variable (Multimedia Appendix 5). The distribution of propensity scores was well balanced between propensity-matched cohorts (Multimedia Appendix 6).

Third, to account for the competing risk of death prior to ICU admission by 48 hours, which occurred in 5 patients, a competing risk regression model (censored at 48 hours following hospital admission) was used in the propensity-matched cohort. Cumulative incidence curves were also estimated. Due to the importance of age as a confounding variable, age-stratified cumulative incidence curves were also generated.

The primary clinical outcome for the above models was the need for ICU admission within 48 hours of hospital admission. This endpoint was chosen clinically as the primary outcome because adherence with anticoagulation best practices is hypothesized to reduce microthrombosis and macrothrombosis events, and minimize progression of the disease and critical illness.

Secondary outcomes of interest for the above models were also evaluated, including all-cause in-hospital mortality, the need for ICU admission at any time during hospitalization, the need for mechanical ventilation, hospital length of stay, and the development of VTE or bleeding complications. Additionally, a binary composite outcome metric was developed and coded as positive if a patient had all-cause in-hospital mortality, required ICU admission, required mechanical ventilation, or required a hospital length of stay greater than 7 days.

The exposure or treatment of interest for the above models was defined as a binary variable if patients received guideline-adherent care on hospital admission.

With regard to confounding variables for the above models, variables known to be associated with the outcome of more severe COVID-19 infection (defined as requiring ICU admission or mechanical ventilation) were included as confounding variables for all analyses. This list of variables was developed by our team of subject matter experts with clinical and research expertise managing patients with COVID-19. All models were risk adjusted to account for patient-level baseline demographics (age, gender, race/ethnicity, English vs non-English speaking, and area deprivation index [a marker of neighborhood socioeconomic status] [20]), the month of admission, in-hospital treatments for COVID-19 (remdesivir, tocilizumab, and steroids), BMI, Elixhauser comorbidity index, the most aberrant vital signs within the first 24 hours of hospital admission (minimum saturation/FiO2 ratio, minimum systolic blood pressure, and maximum respiratory rate), the initial hospital of treatment, and the source of admission (home, emergency department, skilled nursing facility, intrahospital transfer, prescheduled admission for surgery, and admission from a clinic/office appointment).

With regard to subgroup analysis, of the 2503 patients, initial D-dimer, C-reactive protein, creatinine, and absolute neutrophil to absolute lymphocyte ratio data were present for 1181 patients. As these laboratory values have been shown on admission to be predictive of worse clinical outcomes [21-23], a secondary analysis was conducted in these 1181 patients.

With regard to data missingness, overall missingness was low (<2.04% for any individual variable, with 3.9% of patients missing at least one covariate). Given the low rate of missingness, imputation was deemed unnecessary [24].

Statistical analyses were performed using Stata MP, version 16 (StataCorp). Statistical significance was defined as a *P* value <.05.

### Data Availability

The data underlying this article were provided by M Health Fairview (Minneapolis, MN) with permission from M Health Fairview Research and IT. Data will be shared on request to the corresponding author with the permission of M Health Fairview.

## Results

### Patient Characteristics

A total of 2503 patients required in-hospital admission during the study period, with polymerase chain reaction–confirmed COVID-19 (Multimedia Appendix 7). The median patient age was 64.9 years (IQR 48.4-77.7 years). Of the patients, 1180 (47.1%) were male and 262 (10.5%) had in-hospital death. The baseline characteristics of patients who received CPG-adherent (vs nonadherent) care are shown in Table 1. Similarly, the baseline unadjusted clinical outcomes by CPG adherence are shown in Multimedia Appendix 8.

**Table 1:** Patient characteristics.

Characteristic		Did not receive adherent anticoagulation (n=853)	Received adherent anticoagulation (n=1650)	*P* value^a^
Age (years), median (IQR)		60.1 (35.2-75.7)	66.2 (52.7-78.4)	<.001
**Race, n (%)**				.60
	White	476 (55.8)	945 (57.3)	
	Black	117 (13.7)	187 (11.3)	
	Asian	105 (12.3)	211 (12.8)	
	Hispanic	62 (7.3)	114 (6.9)	
	Declined	74 (8.7)	161 (9.8)	
	Other	19 (2.2)	32 (1.9)	
Male, n (%)		350 (41.0)	830 (50.3)	<.001
**Area deprivation index quintile, n (%)**				.58
	0%-19%	168 (19.7)	312 (18.9)	
	20%-39%	256 (30.0)	499 (30.2)	
	40%-59%	231 (27.1)	478 (29.0)	
	60%-79%	114 (13.4)	227 (13.8)	
	80%-100%	84 (9.8)	134 (8.1)	
Non-English Speaking, n (%)		233 (27.3)	477 (28.9)	.40
Elixhauser comorbidity index, median (IQR)		4.0 (1.0-8.0)	5.0 (2.0-8.0)	<.001
BMI, median (IQR)		28.6 (24.6-33.6)	29.8 (25.7-35.4)	<.001
Lowest systolic blood pressure in the first 24 hours (mmHg), median (IQR)		111.0 (98.0-124.0)	113.0 (100.0-127.0)	.01
Highest respiratory rate in the first 24 hours (bpm), median (IQR)		22.0 (18.0-29.0)	24.0 (20.0-32.0)	<.001
Lowest S/F^b^ ratio in the first 24 hours, median (IQR)		438.1 (320.0-459.5)	355.6 (286.4-447.6)	<.001
Initial D-dimer, median (IQR)		1.2 (0.7-2.3)	1.1 (0.6-2.0)	.02
Initial CRP^c^, median (IQR)		64.3 (24.0-125.0)	72.0 (30.8-132.0)	.18
Initial creatinine, median (IQR)		1.0 (0.8-1.4)	1.0 (0.8-1.3)	.39
Initial NLR^d^, median (IQR)		5.1 (3.1-8.4)	4.9 (3.0-8.6)	.97
Received remdesivir, n (%)		203 (24.2)	843 (51.1)	<.001
Received tocilizumab, n (%)		26 (3.0)	90 (5.5)	.007
Received steroids, n (%)		153 (17.9)	575 (34.8)	<.001
**Admission month of 2020, n (%)**				.06
	March	18 (2.1)	20 (1.2)	
	April	64 (7.5)	103 (6.2)	
	May	90 (10.6)	238 (14.4)	
	June	51 (6.0)	103 (6.2)	
	July	65 (7.6)	121 (7.3)	
	August	100 (11.7)	159 (9.6)	
	September	70 (8.2)	112 (6.8)	
	October	143 (16.8)	291 (17.6)	
	November	252 (29.5)	503 (30.5)	
**Implementation site, n (%)**				<.001
	Hospital 0	13 (1.5)	59 (3.6)	
	Hospital 1	14 (1.6)	26 (1.6)	
	Hospital 2	182 (21.3)	363 (22.0)	
	Hospital 3	12 (1.4)	21 (1.3)	
	Hospital 4	18 (2.1)	51 (3.1)	
	Hospital 5	134 (15.7)	268 (16.2)	
	Hospital 6	135 (15.8)	264 (16.0)	
	Hospital 7	56 (6.6)	163 (9.9)	
	Hospital 8	58 (6.8)	50 (3.0)	
	Hospital 9	110 (12.9)	148 (9.0)	
	Hospital 10	72 (8.4)	152 (9.2)	
	Hospital 11	49 (5.7)	85 (5.2)	
**Source of admission, n (%)**				<.001
	Home	391 (46.0)	672 (40.8)	
	Emergency department	374 (44.0)	815 (49.5)	
	Skilled nursing facility	36 (4.2)	62 (3.8)	
	External hospital transfer	33 (3.9)	87 (5.3)	
	Admission for surgery	8 (0.9)	1 (0.1)	
	Clinic	8 (0.9)	11 (0.7)	

^a^The Pearson chi-square test was used to compare categorical and binary variables, and the Wilcoxon rank-sum test was used to compare continuous variables with a skewed distribution.

^b^S/F: oxygen saturation to fraction of inspired oxygen.

^c^CRP: C-reactive protein.

^d^NLR: neutrophil-to-lymphocyte ratio.

### Reach

**Figure 2:**
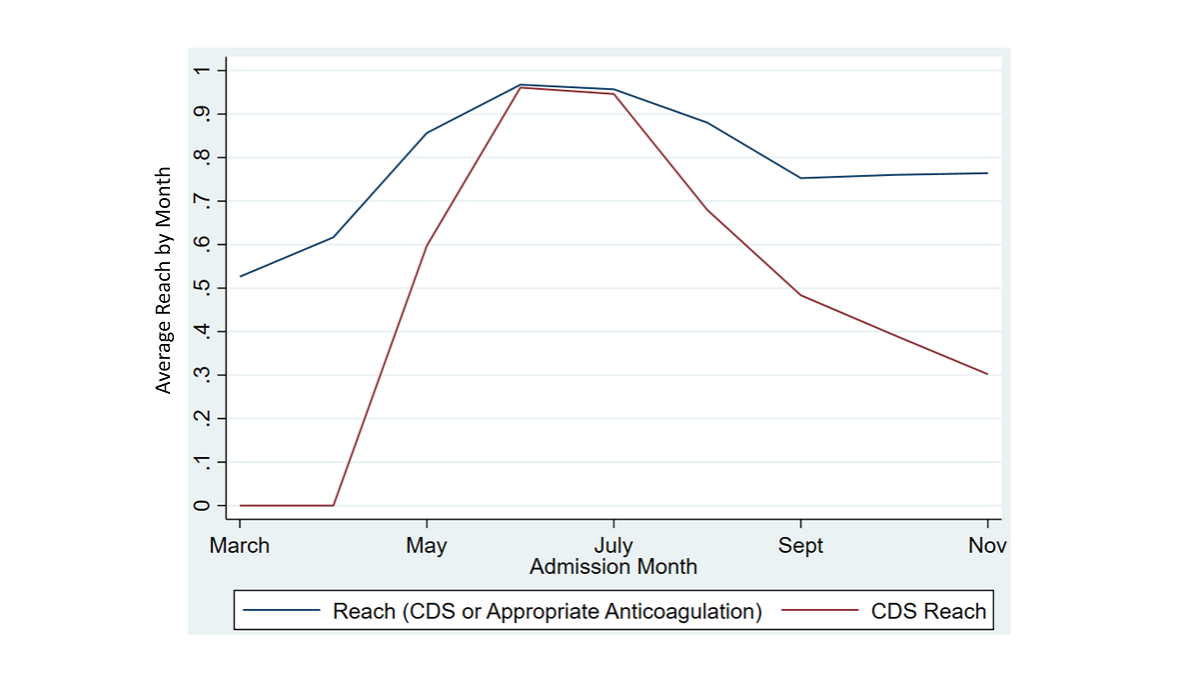
Average implementation reach by month. The blue line represents the combined CPG (patient received adherent anticoagulation) and CDS reach (patient’s ordering providers received the CDS system suggesting adherent anticoagulation) by month. The red line represents only CDS reach. CDS: clinical decision support; CPG, clinical practice guideline.

Figure 2 displays the reach of the CPG by month. Reach was purposefully measured in 2 ways (CPG reach or CDS reach). CPG reach was measured by the percentage of patients who received appropriate anticoagulation. CDS reach was measured by the percentage of patients whose providers received a CDS “reminder” to adhere to the CPG (Figure 2). Figure 2 displays the combined CPG and CDS reach (blue line) compared with CDS reach alone (red line). In an ideal setting, reach (blue line) would approach 1.0 and CDS reach (red line) would approach 0. This would represent the state where all patients are receiving adherence without the need for interruptive CDS and would reflect complete uptake of the CPG by providers. Baseline reach of the anticoagulation CPG in April was approximately 61%. System-wide implementation of a CDS strategy in May resulted in 97% reach. The reduced triggering of CDS after August represents increased ordering of any anticoagulation for COVID-19 patients.

**Figure 3:**
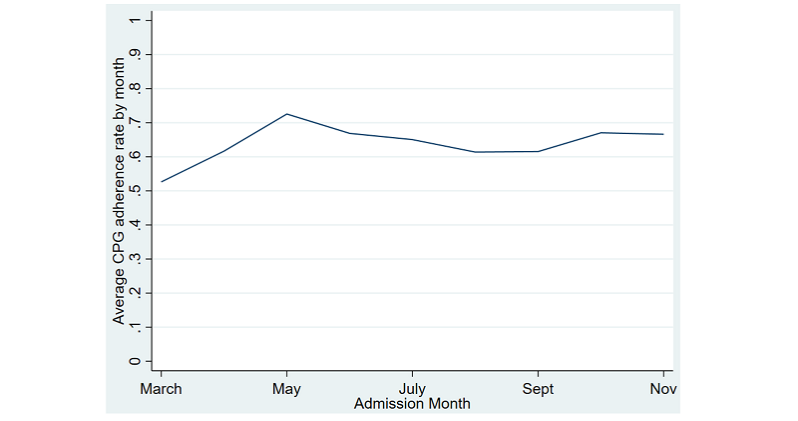
Average implementation reach by month. (A) Average CPG reach by health care system by month. CDS: clinical decision support; CPG: clinical practice guideline.

CPG reach improved following implementation of the CDS system. CPG reach peaked during piloting and scaling of the CDS system with an adherence rate of 74.4%. In the 6 months since scaling, adherence averaged 67% (Figure 3).

### Effectiveness

The *primary hypothesis* tested was if adherence with the anticoagulation CPG on hospital admission was associated with a reduced need for ICU management by 48 hours. Adherence with the anticoagulation CPG was independently associated with reduced need for ICU admission within 48 hours of hospital admission on multivariable logistic regression analysis (odds ratio [OR] 0.39, 95% CI 0.30-0.51; *P*<.001) (Table 2 and Multimedia Appendix 9). In the propensity-matched cohorts, patients who received CPG-adherent care on admission had a 21.46% incidence of ICU admission within 48 hours compared with 34.28% for patients who did not receive adherent care on admission (chi-square *P*<.001; logistic regression OR 0.52; *P*<.001). A time-to-event analysis was also conducted. Patients who received adherent care on admission (vs patients who did not) were more likely to not require ICU admission by 48 hours (log-rank test *P*<.001; Multimedia Appendix 10). Five patients died prior to 48 hours, and thus, to account for this competing risk, a competing risk-regression analysis was conducted. Patients who received adherent care according to the CPG on admission had significantly reduced hazards for ICU admission by 48 hours when accounting for the competing risk of death (subhazard ratio 0.58, *P*<.001). Cumulative incidence functions are provided in Multimedia Appendix 11. As older patients may elect for comfort measures and die in the hospital ward in lieu of aggressive ICU care, an age-stratified cumulative incidence function is provided in Multimedia Appendix 12.

Secondary outcome analysis identified that adherence with the anticoagulation CPG was associated with reduced need for ICU admission at any point during hospitalization (OR 0.53, 95% CI 0.42-0.69; *P*<.001) and reduced all-cause in-hospital mortality (OR 0.67, 95% CI 0.48-0.94; *P*=.02; Table 2). Adherence with the anticoagulation CPG significantly reduced the odds of death, ICU admission, requirement for mechanical ventilation, and hospital length of stay greater than 7 days (OR 0.75, 95% CI 0.60-0.94; *P*=.01). Adherence with the anticoagulation CPG was independently associated with reduced bleeding complications (OR 0.39, 95% CI 0.21-0.72; *P*=.003), but not VTE complications (OR 0.87, 95% CI 0.65-1.17; *P*=.40). Adherence with the anticoagulation CPG was independently associated with an increased hospital length of stay (incident rate ratio [IRR] 1.15, 95% CI 1.08-1.22; *P*<.001). This effect persisted on excluding patients who had in-hospital death (IRR 1.13, 95% CI 1.06-1.2; *P*<.001). None of the other secondary analyses reached statistical significance (Table 2).

**Table 2:** Likelihood of adherence with the clinical practice guideline on multivariable logistic regression.

Variable			Odds ratio for CPG^a^ adherence (vs nonadherence)		95% CI		*P* value		C-statistic^b^
**Model 1: Risk adjustment without initial labs (n=2406)**									
	ICU^c^ admission within 48 hours	0.39		0.30-0.51		<.001		0.87	
	ICU admission	0.53		0.42-0.69		<.001		0.87	
	Required mechanical ventilation	1.18		0.79-1.77		.40		0.93	
	All-cause in-hospital mortality	0.67		0.48-0.94		.02		0.88	
	Composite outcome^d^	0.75		0.60-0.94		.01		0.82	
	VTE^e^ complication	0.87		0.65-1.17		.40		0.79	
	Bleeding complication	0.39		0.21-0.73		.003		0.83	
**Model 2: Risk adjustment including initial labs (n=1181)**									
	ICU admission within 48 hours	0.28		0.19-0.43		<.001		0.90	
	ICU admission	0.44		0.29-0.64		<.001		0.89	
	Required mechanical ventilation	1.20		0.67-2.20		.50		0.94	
	All-cause in-hospital mortality	0.92		0.56-1.52		.70		0.88	
	Composite outcome^d^	0.61		0.42-0.87		.006		0.82	
	VTE complication	1.05		0.64-1.71		.90		0.81	
	Bleeding complication	0.47		0.17-1.26		.10		0.87	

^a^CPG: clinical practice guideline.

^b^C-statistic or concordance statistic was calculated for each model.

^c^ICU: intensive care unit.

^d^Composite outcome is defined as need for ICU admission, mechanical ventilation, all-cause in-hospital mortality, or hospital length of stay greater than 7 days.

^e^VTE: venous thromboembolism.

In the model that included initial D-dimer, C-reactive protein, creatinine, and the neutrophil-to-lymphocyte ratio, adherence with the anticoagulation CPG was independently associated with reduced need for ICU admission within 48 hours of hospital admission (OR 0.28, 95% CI 0.19-0.43; *P*<.001) or the need for ICU admission at any time during hospitalization (OR 0.44, 95% CI 0.29-0.64; *P*<.001) (Table 2). Adherence with the anticoagulation CPG significantly reduced the odds of death, ICU admission, requirement for mechanical ventilation, or hospital length of stay greater than 7 days (OR 0.61, 95% CI 0.42-0.87; *P*=.006). Adherence with the anticoagulation CPG was independently associated with an increased hospital length of stay (IRR 1.12, 95% CI 1.03-1.22; *P*=.008). This effect persisted on excluding patients who had in-hospital death (IRR 1.1, 95% CI 1.004-1.2; *P*=.04). None of the other secondary analyses reached statistical significance (Table 2).

### Adoption

To investigate adoption rates across the system, we evaluated adoption by hospital. Our system includes 12 hospitals, 2 university settings that include resident and fellow trainees, 2 COVID-19 cohorted hospitals [25] staffed by attending physicians and advanced practice providers, and 8 community hospitals staffed by attending physicians and advanced practice providers. Adoption was the highest at the COVID-19 cohorted hospitals and lowest at the university hospitals (Multimedia Appendix 13). Variability was similarly noted across nursing units (Multimedia Appendix 14). No discernable difference was noted in adoption analyses performed by patient race/ethnicity, encounter type, or investigation for COVID-19 status on admission (versus known COVID-19).

### Implementation and Maintenance

Adherence was evaluated in the context of CDS. Adherence when CDS was delivered was 70% as compared to 62% without CDS (OR 1.43, 95% CI 1.2-1.7; *P*<.001).

The CDS 5 Rights Framework recommends delivery of CDS at the “right” time in workflow. Four passive CDS elements were included to facilitate CPG adherence within various areas of the EHR and clinical workflow. For example, passive CDS was delivered within EHR navigators used during the admission, discharge, or transfer (ADT) workflow, during the rounding navigator, and within the general EHR order environment. Adherence with anticoagulation was the highest when these passive elements were integrated within the admission (75%), rounding (75%), or transfer (80%) navigators (Multimedia Appendix 15) than when outside of an EHR care navigator (57%).

We then sought to investigate the relationship between adherence and passive versus interruptive CDS intervention formats. Overall, 1423 (56.9%) patients had no CDS elements that were passive or interruptive. On the other hand, 699 (27.9%) patients had passive-only CDS delivered to providers, 111 (4.4%) patients had interruptive-only CDS delivered to providers, and 270 (10.8%) patients had a combination of passive and interruptive CDS delivered to providers. The combination of passive CDS and interruptive alerts was associated with the highest adherence with the anticoagulation CPG (Multimedia Appendix 16).

Variation in adherence was noted across baseline risk groups. Patients in the moderate-risk group were less likely to receive adherent care (606/1016, 59.6%) compared with patients in the high- (145/223, 65%) and low-risk groups (899/1264, 71%). Following implementation, wash-out maintenance stabilized at 67% in months 5 to 6 (October to November 2020).

## Discussion

### Real-World Application of the LHS

This study represents the completed iteration of a continuous LHS cycle [26]. Adherence with the anticoagulation CPG was associated with significantly improved clinical outcomes. Adoption improved following the delivery of the CPG within a CDS system. Despite these improvements, variation was found in adoption across hospitals and units. Adoption was the highest at hospitals specializing in treating patients with COVID-19 and was the lowest in tertiary academic hospitals. An evaluation of CDS delivery methods identified that the combination of passive and interruptive alerts was associated with the highest adherence rate.

This study provides an important and early example of the real-world application of the LHS during COVID-19, a period with surged clinical resources and uncertain evidence base. Critical to our success was the early development of a COVID-19 data mart that included highly granular structured and unstructured patient-level data. Integration with an EHR analysis solution (LogicStream Health) facilitated near real-time evaluation of CDS alert activities by providers.

Our health care system has a rigorous and validated protocol for the development, implementation, scaling, and evaluation of user-centered CDS systems with over 20 use cases implemented each year overseen by various enterprise CDS committees. Typically, the process of development, implementation, and scaling requires months, and in this case, it occurred in a matter of weeks. COVID-19 provided a heightened sense of urgency and purpose in health care research and quality improvement that resulted in rapid progress in CDS development. The dedicated EBM team facilitated prompt CPG updates in response to rapidly changing evidence. Augmented stakeholder engagement and buy-in from the informatics development team were also critical elements for success. The combination of expedited access to fully preprocessed and analyzable EHR data updated daily along with extraordinary team engagement and stakeholder support were critical for rapid implementation.

Despite these successes, the room for optimization was identified from this analysis. First, in an attempt to minimize alert fatigue, the CDS system was initially designed to only trigger for patients with COVID-19 but not on anticoagulation. While it was successful for the months of June and July in achieving near 95% reach, based on the data presented in this study, we hypothesize that providers became comfortable attempting to order anticoagulation independent of the order set, resulting in patients being on incorrect anticoagulation and preventing corrective triggering of the CDS system. Others have shared this experience, where an attempt to develop a user-centered CDS system minimizing alerts resulted in a system that was overly passive and could not change behavior [27]. Despite all the negative press for interruptive alerts, we were surprised that interruptive alerts and the combination of interruptive alerts and passive CDS were associated with improved adherence compared to passive CDS alone. It is possible due to the COVID-19 pandemic and the augmented sense of unity and purpose between clinicians and quality improvement researchers that interruptive alerts were received more favorably.

Second, we identified a large variation in adoption across hospitals and nursing units. Our academic health system is unique in the sense that we created specialty cohorted hospitals for COVID-19 patients [25] needing care across our academic health medical center, which was staffed by attending clinicians well versed with institutional guidelines. Despite the availability of the same resources at all sites, adoption of the CPG via the use of the CDS system was much lower at noncohorted sites. Specifically, we identified that adoption was very poor at university sites where the majority of orders are placed by house staff compared with advanced practice providers at other sites.

Third, following each LHS evaluation cycle (practice to data), it is imperative that positive findings are disseminated widely. We were surprised that adherence did not improve following our interim effectiveness analysis in August 2020, which identified a significant and independent improvement in clinical outcomes with anticoagulation. The unified theory of acceptance and use of technology posits that a key construct affecting technology use intention (in this case, using the CDS system) is performance expectancy [28]. Essentially, if the provider believes that the CDS system will improve patient outcomes, they will have higher intentions to use it. In response to this RE-AIM evaluation, we will pilot an education intervention, provide continuing medical education (CME) credit as an added incentive for participation at sites with lower adoption, and assess the impact at our next Plan-Do-Study-Act (PDSA) cycle.

In the early phase of the pandemic, there was widespread confusion about the underlying pathogenic mechanism and its implications for patient outcomes, leading to highly variable practice in the medical community. To date, limited data exist on the management approach for COVID-19–associated coagulopathy, particularly in high-risk critically ill populations and for patients who are either managed as outpatients or posthospital discharge patients [29,30]. At the time of writing this manuscript, there were 147 (32 from the United States) randomized trials ongoing or recently completed to assess different anticoagulation approaches in COVID-19 [31]. Since the completion of this evaluation in December 2020, results of multiple COVID-19 anticoagulation randomized trials have since been published. While a formal review of the literature was outside the scope of this study, controversy persists as 2 recently published open-label randomized trials offered conflicting evidence, with one suggesting a lack of benefit from therapeutic-dose anticoagulation in critically ill patients [10] and the other showing significant improvement in survival and increased organ support-free days in noncritically ill patients [9]. However, both these studies had multiple limitations [32] and both evaluated therapeutic anticoagulation doses, whereas our consensus guideline includes a tiered approach including intermediate anticoagulation for specific high-risk subsets. During the study period, our tiered approach recommended an intermediate dose for critically ill patients. In March 2021, the INSPIRATION trial reported its findings and did not identify an advantage of intermediate-dose versus prophylactic-dose anticoagulation for critically ill patients with COVID-19 [33]. Although this was an observational study, it provides additional support that adherence with a tiered approach for anticoagulation in patients with COVID-19 is associated with improved clinical outcomes and, in our health care setting, reduced bleeding complications. Interestingly, we noted that adherence was associated with reduced bleeding but not VTE complications. This may suggest that this approach does not impact large vessel VTE, but improved outcomes overall suggest that patients may be developing fewer microvessel thrombi, causing less systemic complications that typically lead to ICU admissions and adverse outcomes.

COVID-19 is a global emergency; given the lack of robust/consistent guidelines from leading societies, institutions had to develop a local approach to create a uniform plan of care and an approach for its implementation. This is specifically problematic in larger systems with multiple hospitals. Our health system was particularly vulnerable to this issue owing to significant heterogeneity resulting from a recent merger (different instances of the same EHR, heterogeneous administrative policies, and site-specific management protocols). As described above, there were concerns for an increased risk of bleeding, and many individual practitioners in our system were apprehensive to order anticoagulation in patients with COVID-19, leading to variable VTE prevention strategies and adverse patient outcomes.

Our study has several limitations. First, our institutional preference for implementation evaluation is typically a mixed-methods approach. However, due to contact precautions surrounding COVID-19 and significantly increased provider workload, it was not feasible to perform a qualitative analysis of staff. Thus, this represents a quantitative-only approach, which may not fully discern specific trends. To expand on hypotheses that arose from this research, a future direction includes a voluntary survey of health care providers (initiated on December 14, 2020) surrounding their familiarity with COVID-19 institutional guidelines and their experiences interacting with the CDS system. Survey question development was guided by unified theory of acceptance and use of technology constructs for technology acceptance. Additionally, while we identified an association with adherence and the effectiveness of anticoagulation, it is important to not misconstrue this analysis. We did not evaluate anticoagulation versus no anticoagulation and the association with outcomes, but rather we evaluated adherence with the guideline versus nonadherence with the guideline. Thus, nonadherent patients could have been receiving either more or less aggressive anticoagulation than the comparison group. In our institution, adherence was associated with improved clinical outcomes; however, this may not be generalizable to other institutions with different baseline cultural practices for anticoagulation management. VTE and bleeding complications were extracted using structured EHR data. These events may be underreported. Our relatively small cohort size (n=2503) and single health care system represent additional limitations of the study.

### Conclusion

This study provides an early example of the real-world application of the LHS during COVID-19. With or without a pandemic, there is a need for the implementation of evidence-based practice that is most up-to-date. Traditionally, the largest barrier to this effort has been the need for making major changes in the workflow. With the widespread use of EHRs and increasing consolidation of health care systems, the application of a CPG through the use of a CDS system can offer an easy tool for implementation without adding confusion related to workflow changes, thus bringing uniformity in care at every level in the system and influencing the quality of care and patient outcomes.

## Appendix

Multimedia Appendix 1 M Health Fairview COVID-19 anticoagulation clinical practice guideline.

Multimedia Appendix 2 Process map of the M Health Fairview clinical decision support system.

Multimedia Appendix 3 Screenshots of the COVID-19 anticoagulation clinical decision support system's passive and interruptive elements.

Multimedia Appendix 4 Screenshots of the COVID-19 anticoagulation clinical decision support system's surveillance of appropriate anticoagulation prophylaxis.

Multimedia Appendix 5 Propensity score standardized differences prior to and after matching.

Multimedia Appendix 6 Propensity score distribution in propensity-matched cohorts. Blue/untreated refers to the propensity-matched patient cohort that did not receive CPG adherent care on hospital admission. Red/treated refers to the propensity-matched patient cohort that did receive CPG adherent care on hospital admission. CPG: clinical practice guideline.

Multimedia Appendix 7 Study diagram for the selection of patients from the COVID-19 inpatient database.

Multimedia Appendix 8 Clinical outcomes.

Multimedia Appendix 9 Full model output for multivariable logistic regression evaluating the association of adherence with the clinical practice guideline on hospital admission with the need for intensive care within 48 hours.

Multimedia Appendix 10 Kaplan-Meier failure estimates (for intensive care unit admission by 48 hours). Kaplan-Meier failure estimates for patients who received CPG adherent care (red line) versus those who did not receive CPG adherent care (blue line). CPG: clinical practice guideline.

Multimedia Appendix 11 Cumulative incidence function of intensive care unit (ICU) admission by 48 hours. Cumulative incidence function of ICU admission by 48 hours for patients who received CPG adherent care (red line) versus those who did not receive CPG adherent care (blue line). CPG: clinical practice guideline.

Multimedia Appendix 12 Age-stratified cumulative incidence function of intensive care unit (ICU) admission by 48 hours. Age-stratified cumulative incidence function of ICU admission by 48 hours for patients who received CPG adherent care versus those who did not receive CPG adherent care. CPG: clinical practice guideline.

Multimedia Appendix 13 Mean adoption by implementation site.

Multimedia Appendix 14 Mean adoption by nursing unit.

Multimedia Appendix 15 Adherence with the anticoagulation CPG by passive CDS elements. CDS: clinical decision support; CPG: clinical practice guideline.

Multimedia Appendix 16 Adherence with the anticoagulation CPG by CDS type. CDS: clinical decision support; CPG: clinical practice guideline.

## Abbreviations

CDS: clinical decision support
CPG: clinical practice guideline
EBM: evidence-based medicine
EHR: electronic health record
ICU: intensive care unit
IRR: incident rate ratio
LHS: learning health system
OR: odds ratio
RE-AIM: Reach, Effectiveness, Adoption, Implementation, and Maintenance
VTE: venous thromboembolism

## References

[1] Mei H, Luo L, Hu Y (2020). Thrombocytopenia and thrombosis in hospitalized patients with COVID-19. J Hematol Oncol.

[2] Kyriakoulis KG, Kokkinidis DG, Kyprianou IA, Papanastasiou CA, Archontakis-Barakakis P, Doundoulakis I, Bakoyiannis C, Giannakoulas G, Palaiodimos L (2021). Venous thromboembolism in the era of COVID-19. Phlebology.

[3] Atallah B, Mallah S, AlMahmeed W (2020). Anticoagulation in COVID-19. Eur Heart J Cardiovasc Pharmacother.

[4] Klok F, Kruip M, van der Meer N, Arbous M, Gommers D, Kant K, Kaptein F, van Paassen J, Stals M, Huisman M, Endeman H (2020). Incidence of thrombotic complications in critically ill ICU patients with COVID-19. Thromb Res.

[5] Bikdeli B, Madhavan MV, Jimenez D, Chuich T, Dreyfus I, Driggin E, Nigoghossian CD, Ageno W, Madjid M, Guo Y, Tang LV, Hu Y, Giri J, Cushman M, Quéré I, Dimakakos EP, Gibson CM, Lippi G, Favaloro EJ, Fareed J, Caprini JA, Tafur AJ, Burton JR, Francese DP, Wang EY, Falanga A, McLintock C, Hunt BJ, Spyropoulos AC, Barnes GD, Eikelboom JW, Weinberg I, Schulman S, Carrier M, Piazza G, Beckman JA, Steg PG, Stone GW, Rosenkranz S, Goldhaber SZ, Parikh SA, Monreal M, Krumholz HM, Konstantinides SV, Weitz JI, Lip GY, Global COVID-19 Thrombosis Collaborative Group‚ Endorsed by the ISTH‚ NATF‚ ESVM‚the IUA‚ Supported by the ESC Working Group on Pulmonary CirculationRight Ventricular Function (2020). COVID-19 and Thrombotic or Thromboembolic Disease: Implications for Prevention, Antithrombotic Therapy, and Follow-Up: JACC State-of-the-Art Review. J Am Coll Cardiol.

[6] Tang N, Bai H, Chen X, Gong J, Li D, Sun Z (2020). Anticoagulant treatment is associated with decreased mortality in severe coronavirus disease 2019 patients with coagulopathy. J Thromb Haemost.

[7] Paranjpe I, Fuster V, Lala A, Russak AJ, Glicksberg BS, Levin MA, Charney AW, Narula J, Fayad ZA, Bagiella E, Zhao S, Nadkarni GN (2020). Association of Treatment Dose Anticoagulation With In-Hospital Survival Among Hospitalized Patients With COVID-19. J Am Coll Cardiol.

[8] Obi AT, Tignanelli CJ, Jacobs BN, Arya S, Park PK, Wakefield TW, Henke PK, Napolitano LM (2019). Empirical systemic anticoagulation is associated with decreased venous thromboembolism in critically ill influenza A H1N1 acute respiratory distress syndrome patients. J Vasc Surg Venous Lymphat Disord.

[9] The ATTACC, ACTIV-4a, and REMAP-CAP Investigators (2021). Therapeutic Anticoagulation with Heparin in Noncritically Ill Patients with Covid-19. N Engl J Med.

[10] The REMAP-CAP, ACTIV-4a, and ATTACC Investigators (2021). Therapeutic Anticoagulation with Heparin in Critically Ill Patients with Covid-19. N Engl J Med.

[11] Macheel C, Reicks P, Sybrant C, Evans C, Farhat J, West MA, Tignanelli CJ (2020). Clinical Decision Support Intervention for Rib Fracture Treatment. J Am Coll Surg.

[12] Nguyen AS, Yang S, Thielen BV, Techar K, Lorenzo RM, Berg C, Palmer C, Gipson JL, West MA, Tignanelli CJ (2020). Clinical Decision Support Intervention and Time to Imaging in Older Patients with Traumatic Brain Injury. J Am Coll Surg.

[13] Teoh D, Vogel RI, Langer A, Bharucha J, Geller MA, Harwood E, Kulasingam S, Melton GB (2019). Effect of an Electronic Health Record Decision Support Alert to Decrease Excess Cervical Cancer Screening. J Low Genit Tract Dis.

[14] Khairat S, Marc D, Crosby W, Al Sanousi A (2018). Reasons For Physicians Not Adopting Clinical Decision Support Systems: Critical Analysis. JMIR Med Inform.

[15] Glasgow RE, Vogt TM, Boles SM (1999). Evaluating the public health impact of health promotion interventions: the RE-AIM framework. Am J Public Health.

[16] Ingraham N, Tignanelli C (2020). Fact Versus Science Fiction: Fighting Coronavirus Disease 2019 Requires the Wisdom to Know the Difference. Crit Care Explor.

[17] University of Minnesota Evidence Based Medicine Homepage for COVID-19.

[18] Wang J, Hajizadeh N, Moore EE, McIntyre RC, Moore PK, Veress LA, Yaffe MB, Moore HB, Barrett CD (2020). Tissue plasminogen activator (tPA) treatment for COVID-19 associated acute respiratory distress syndrome (ARDS): A case series. J Thromb Haemost.

[19] Tignanelli CJ, Gipson J, Nguyen A, Martinez R, Yang S, Reicks PL, Sybrant C, Roach R, Thorson M, West MA (2020). Implementation of a Prophylactic Anticoagulation Guideline for Patients with Traumatic Brain Injury. Jt Comm J Qual Patient Saf.

[20] Ingraham N, Purcell L, Karam B, Dudley RA, Usher MG, Warlick CA, Allen ML, Melton GB, Charles A, Tignanelli CJ (2021). Racial and Ethnic Disparities in Hospital Admissions from COVID-19: Determining the Impact of Neighborhood Deprivation and Primary Language. J Gen Intern Med.

[21] Ponti G, Maccaferri M, Ruini C, Tomasi A, Ozben T (2020). Biomarkers associated with COVID-19 disease progression. Crit Rev Clin Lab Sci.

[22] Lusczek E, Ingraham N, Karam B, Proper J, Siegel L, Helgeson ES, Lotfi-Emran S, Zolfaghari EJ, Jones E, Usher MG, Chipman JG, Dudley RA, Benson B, Melton GB, Charles A, Lupei MI, Tignanelli CJ (2021). Characterizing COVID-19 clinical phenotypes and associated comorbidities and complication profiles. PLoS One.

[23] Ingraham NE, Lotfi-Emran S, Thielen BK, Techar K, Morris RS, Holtan SG, Dudley RA, Tignanelli CJ (2020). Immunomodulation in COVID-19. The Lancet Respiratory Medicine.

[24] Jakobsen JC, Gluud C, Wetterslev J, Winkel P (2017). When and how should multiple imputation be used for handling missing data in randomised clinical trials - a practical guide with flowcharts. BMC Med Res Methodol.

[25] Robbins A, Beilman GJ, Amdahl B, Welton M, Tignanelli C, Olson AP, Chipman JG (2020). Transforming a Long-Term Acute Care Hospital into a COVID-19-Designated Hospital. Surg Infect (Larchmt).

[26] Friedman CP, Rubin JC, Sullivan KJ (2017). Toward an Information Infrastructure for Global Health Improvement. Yearb Med Inform.

[27] Mann D, Hess R, McGinn T, Mishuris R, Chokshi S, McCullagh L, Smith PD, Palmisano J, Richardson S, Feldstein DA (2019). Adaptive design of a clinical decision support tool: What the impact on utilization rates means for future CDS research. Digit Health.

[28] Venkatesh V, Morris MG, Davis GB, Davis FD (2003). User Acceptance of Information Technology: Toward a Unified View. MIS Quarterly.

[29] Patell R, Midha S, Kimani S, Martin R, Neparidze N, Jaglal M, Freed J, Key NS (2020). Variability in Institutional Guidance for COVID-19-Associated Coagulopathy in the United States. Thromb Haemost.

[30] Dicks AB, Weinberg I (2021). Further Evidence Supporting the Use of Prophylactic Anticoagulation in Hospitalized Patients With COVID-19. JAMA Netw Open.

[31] Search results. ClinicalTrials.gov.

[32] ten Cate H (2021). Surviving Covid-19 with Heparin?. N Engl J Med.

[33] Sadeghipour P, Talasaz A, Rashidi F, Sharif-Kashani B, Beigmohammadi MT, Farrokhpour M, Sezavar SH, Payandemehr P, Dabbagh A, Moghadam KG, Jamalkhani S, Khalili H, Yadollahzadeh M, Riahi T, Rezaeifar P, Tahamtan O, Matin S, Abedini A, Lookzadeh S, Rahmani H, Zoghi E, Mohammadi K, Sadeghipour P, Abri H, Tabrizi S, Mousavian SM, Shahmirzaei S, Bakhshandeh H, Amin A, Rafiee F, Baghizadeh E, Mohebbi B, Parhizgar SE, Aliannejad R, Eslami V, Kashefizadeh A, Kakavand H, Hosseini SH, Shafaghi S, Ghazi SF, Najafi A, Jimenez D, Gupta A, Madhavan MV, Sethi SS, Parikh SA, Monreal M, Hadavand N, Hajighasemi A, Maleki M, Sadeghian S, Piazza G, Kirtane AJ, Van Tassell BW, Dobesh PP, Stone GW, Lip GYH, Krumholz HM, Goldhaber SZ, Bikdeli B, INSPIRATION Investigators (2021). Effect of Intermediate-Dose vs Standard-Dose Prophylactic Anticoagulation on Thrombotic Events, Extracorporeal Membrane Oxygenation Treatment, or Mortality Among Patients With COVID-19 Admitted to the Intensive Care Unit: The INSPIRATION Randomized Clinical Trial. JAMA.

