# Supplementary figures and images for: Implementation of an Anticoagulation Practice Guideline for COVID-19 via a Clinical Decision Support System in a Large Academic Health System and Its Evaluation: Observational Study
Source: JMIR Med Inform. 2021 Nov 18;9(11):e30743. doi: 10.2196/30743 (PMC8604256; doi:10.2196/30743)

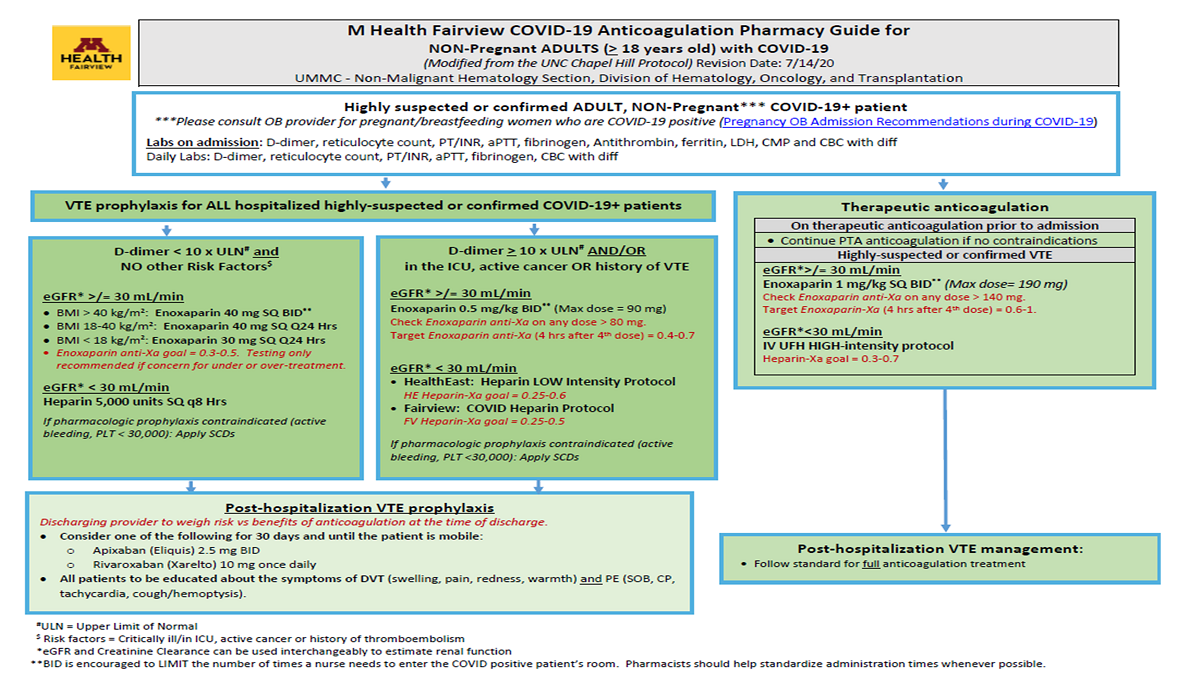

Supplement: Multimedia Appendix 1 [file medinform_v9i11e30743_app1.png]

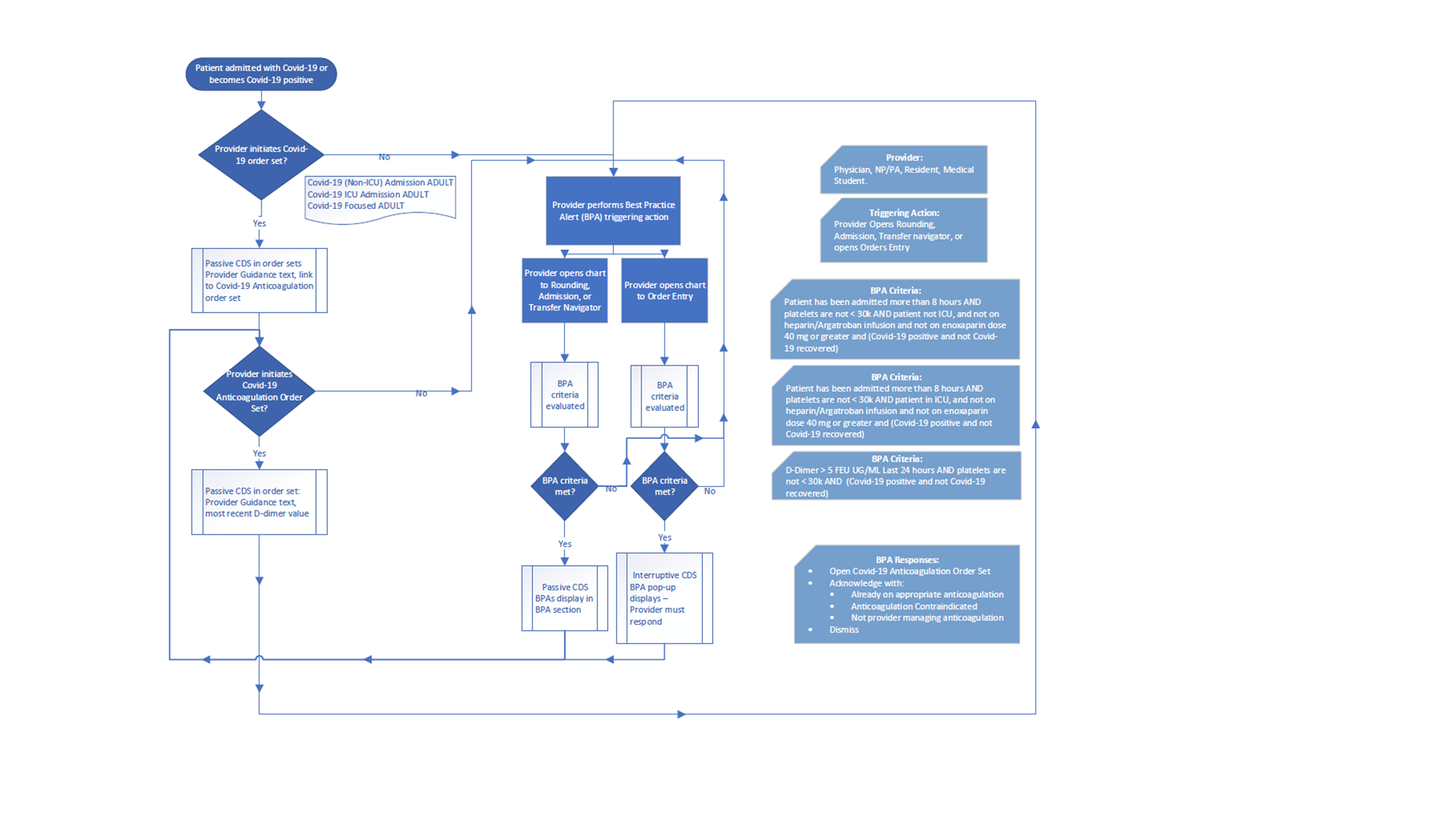

Supplement: Multimedia Appendix 2 [file medinform_v9i11e30743_app2.png]

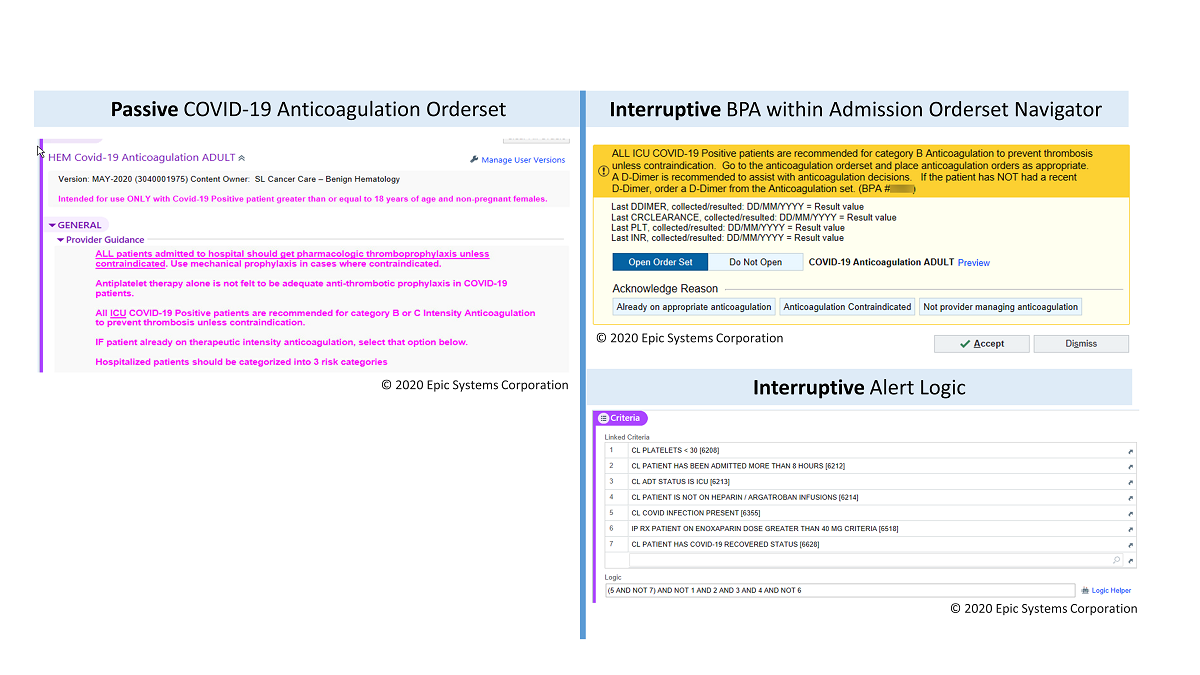

Supplement: Multimedia Appendix 3 [file medinform_v9i11e30743_app3.png]

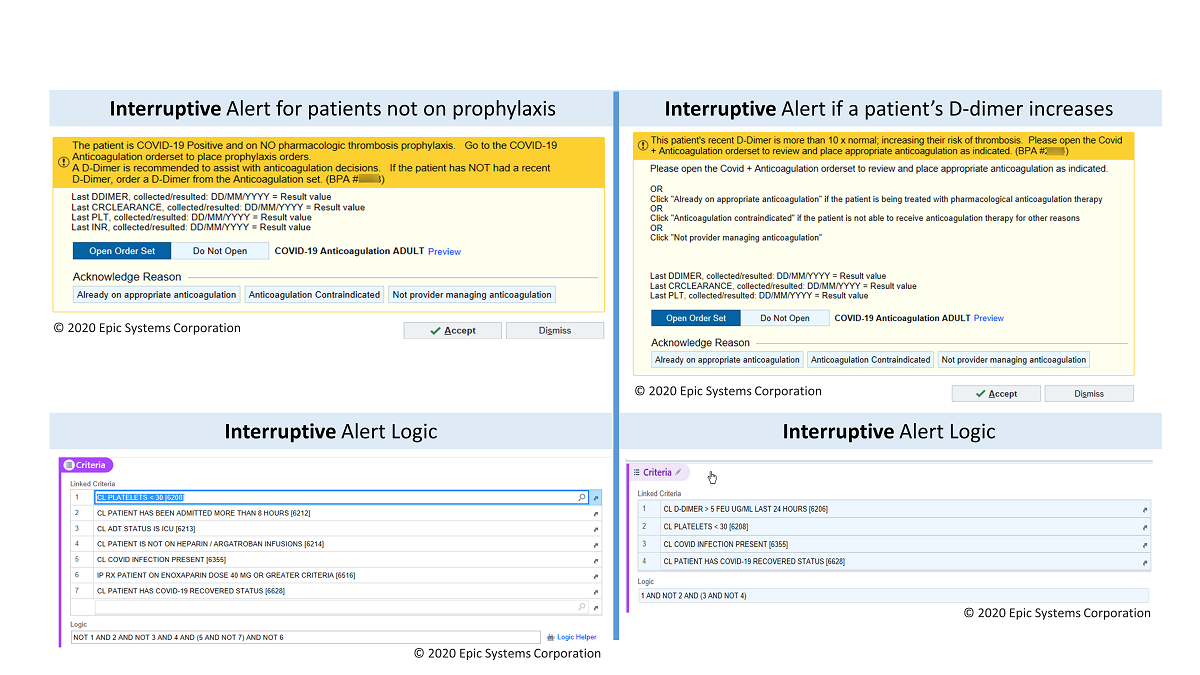

Supplement: Multimedia Appendix 4 [file medinform_v9i11e30743_app4.png]

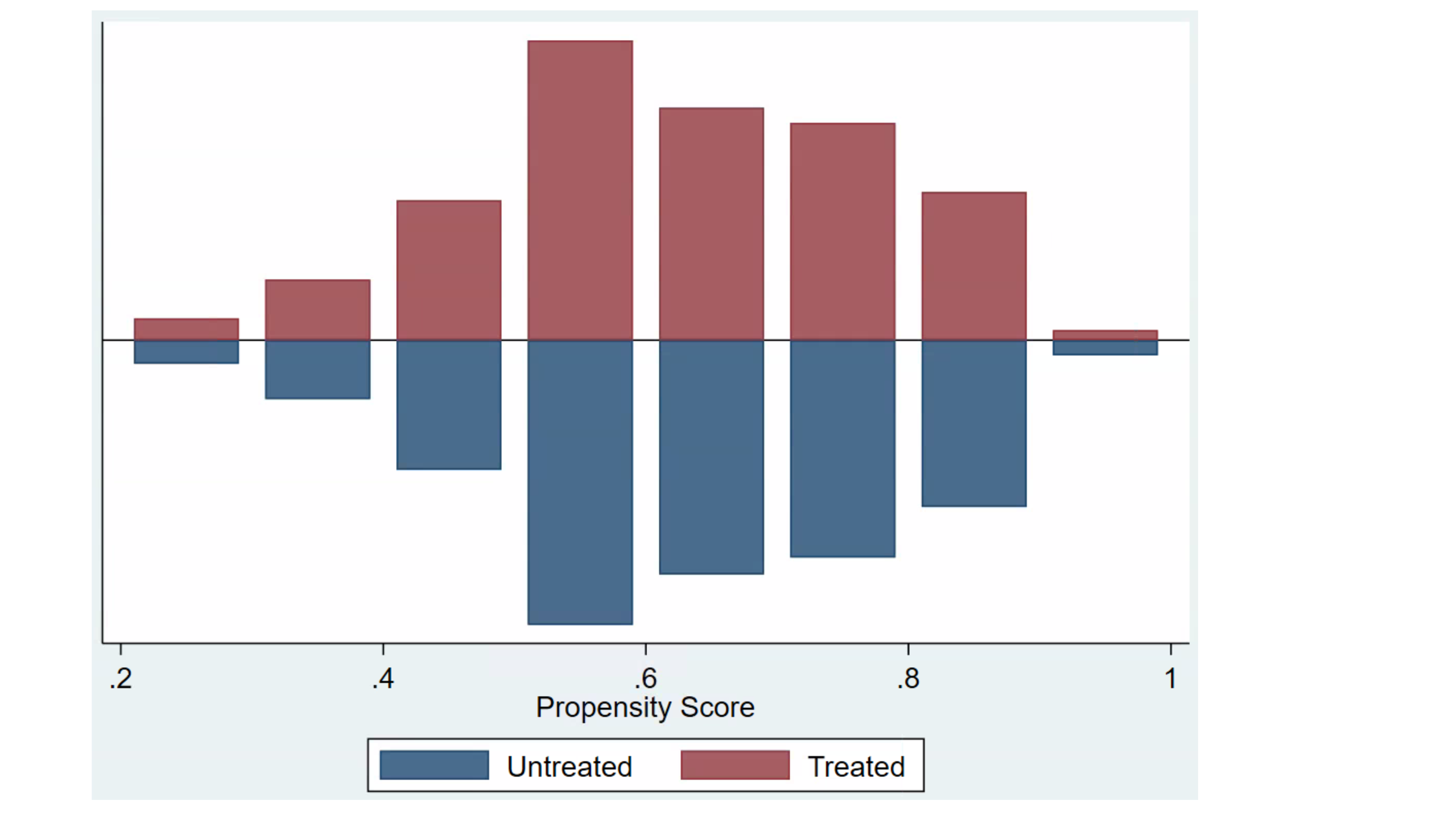

Supplement: Multimedia Appendix 6 [file medinform_v9i11e30743_app6.png]

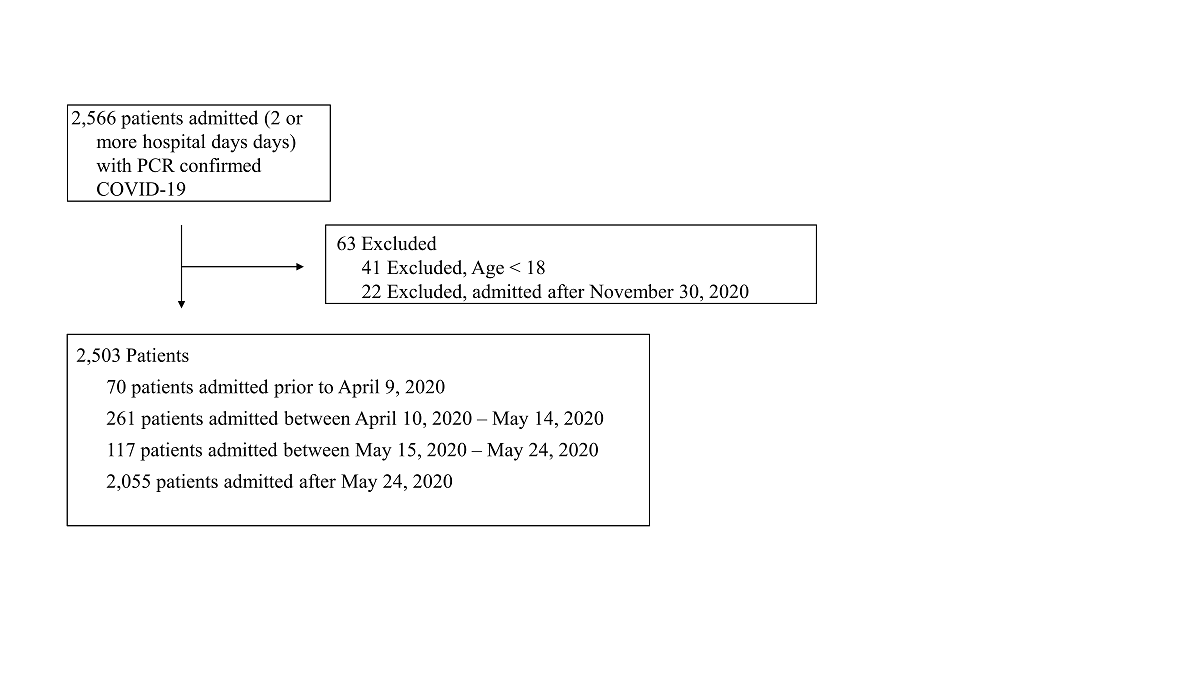

Supplement: Multimedia Appendix 7 [file medinform_v9i11e30743_app7.png]

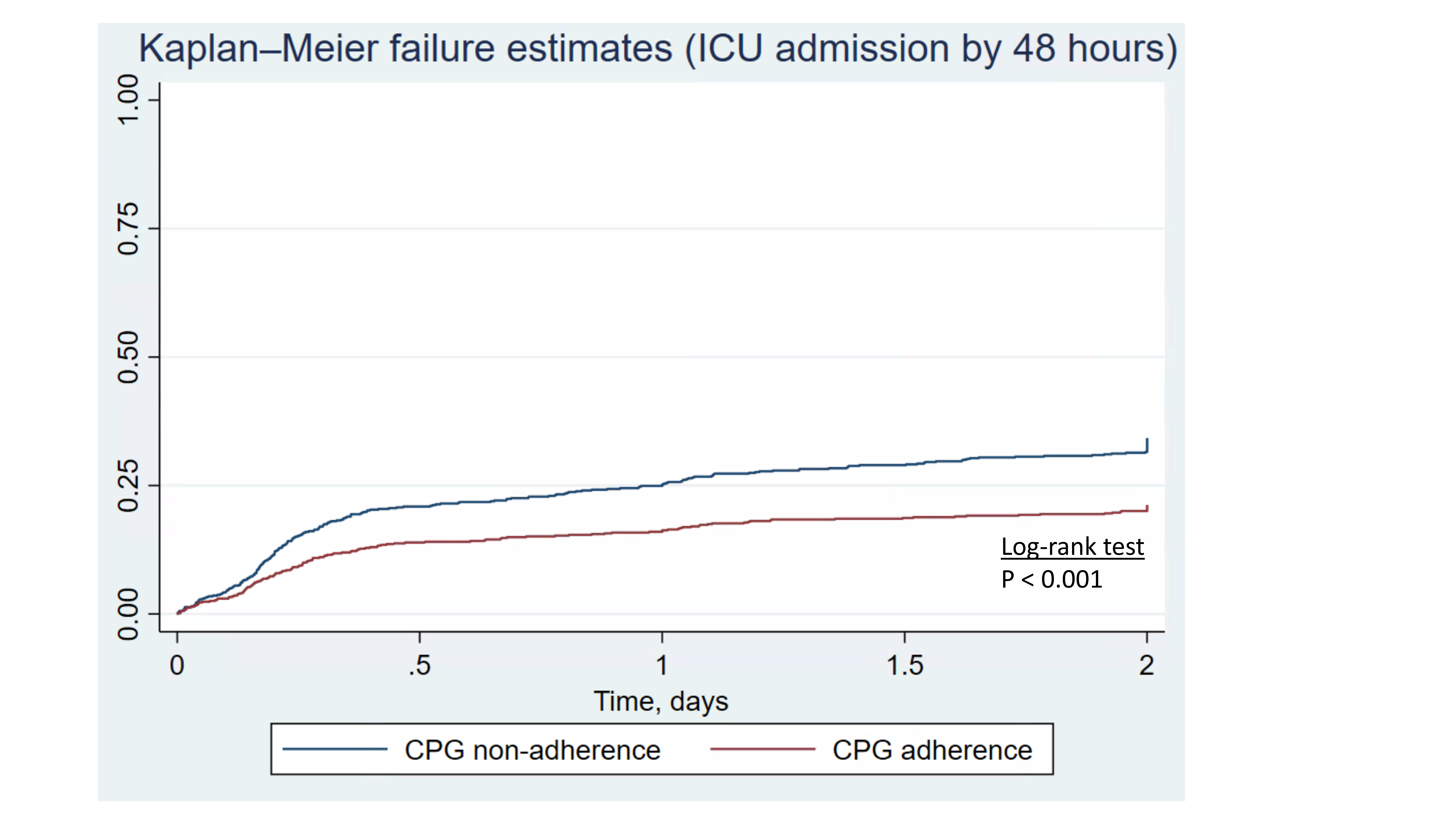

Supplement: Multimedia Appendix 10 [file medinform_v9i11e30743_app10.png]

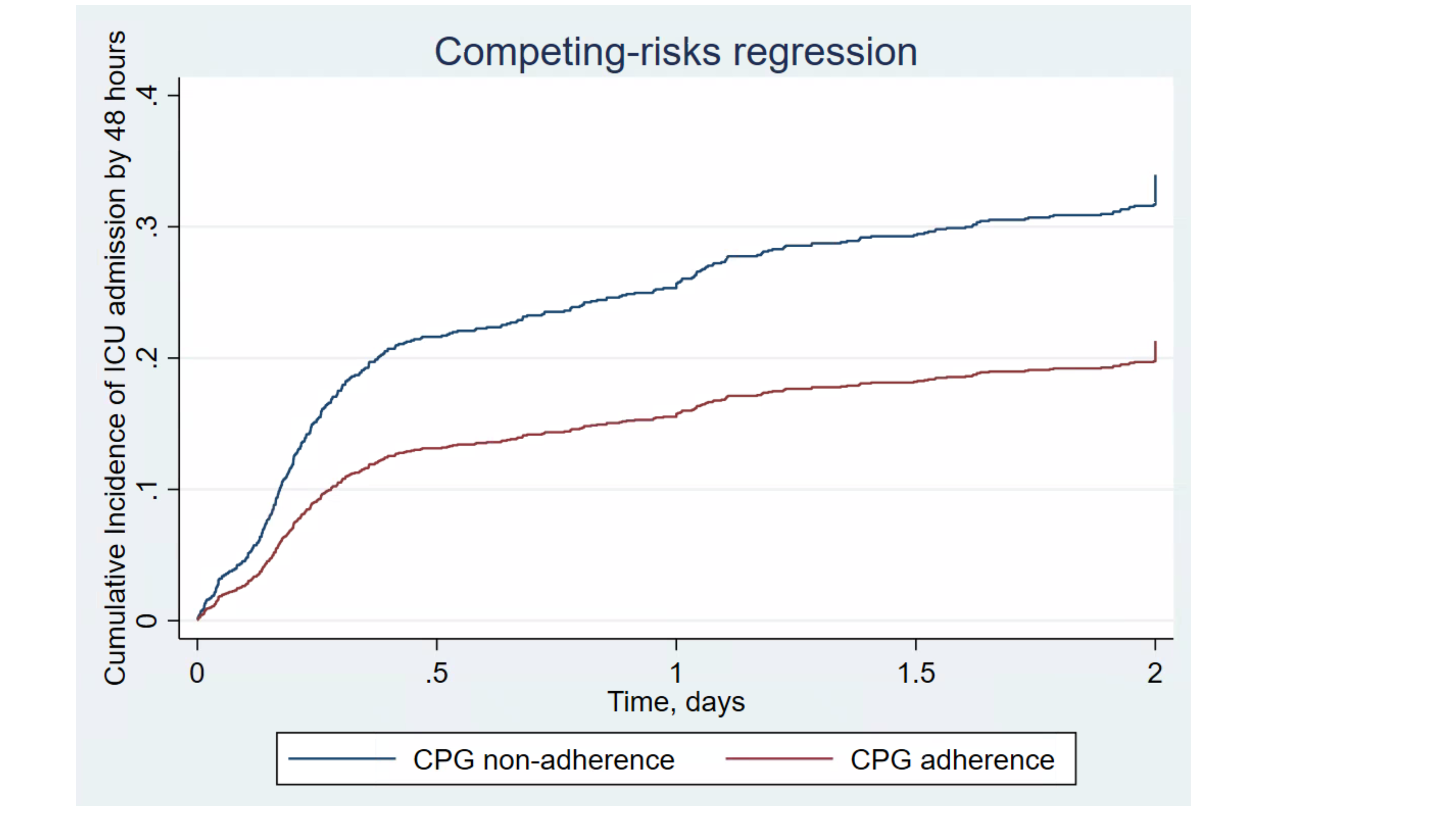

Supplement: Multimedia Appendix 11 [file medinform_v9i11e30743_app11.png]

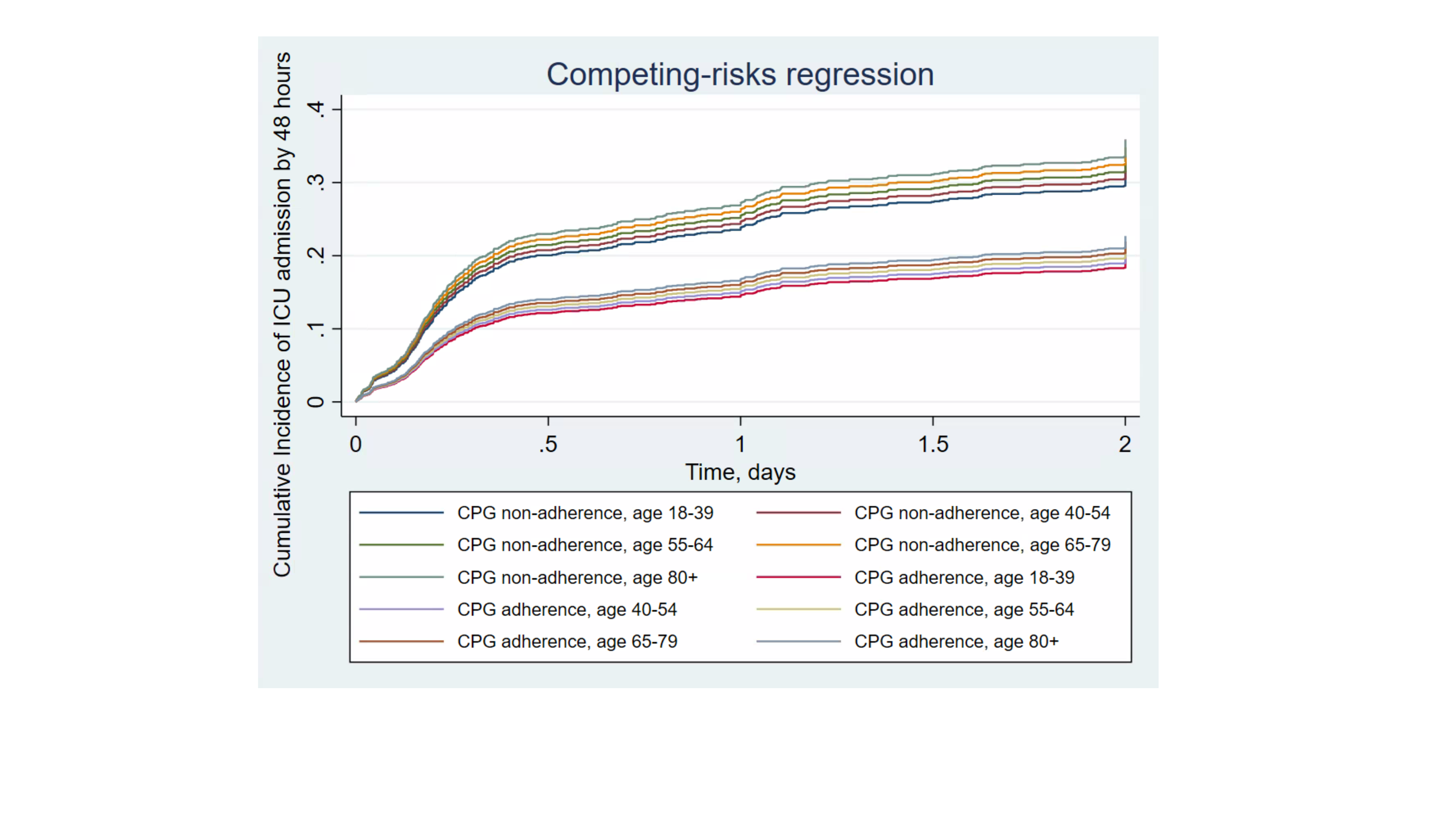

Supplement: Multimedia Appendix 12 [file medinform_v9i11e30743_app12.png]

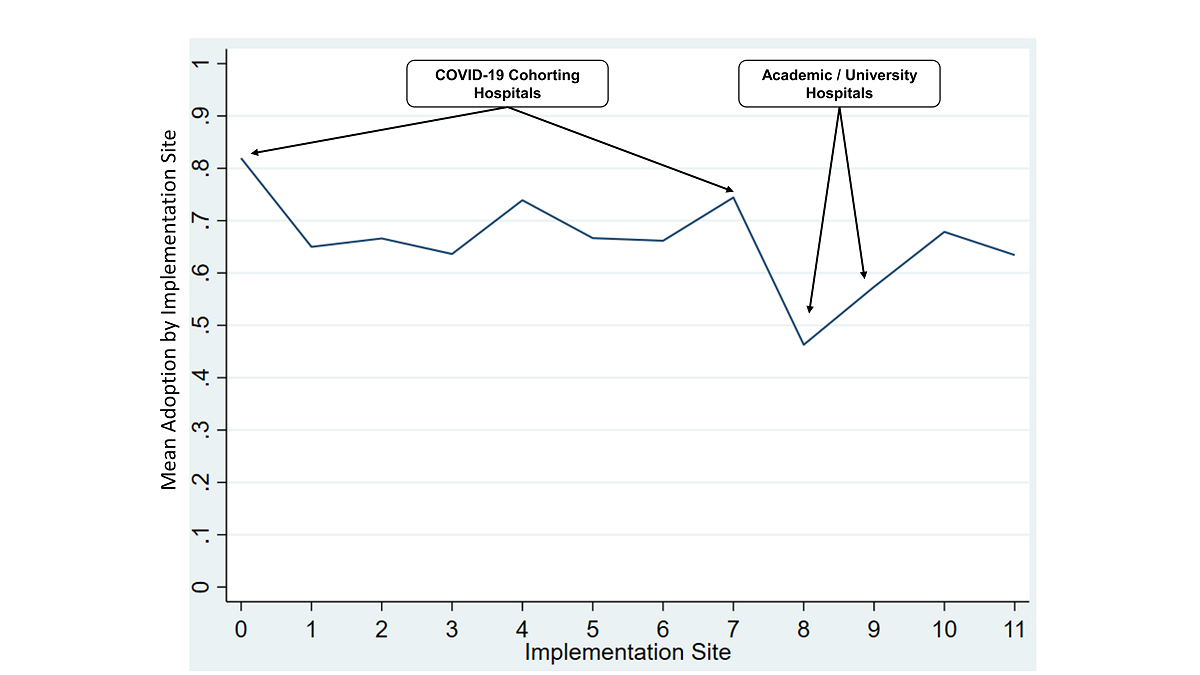

Supplement: Multimedia Appendix 13 [file medinform_v9i11e30743_app13.png]

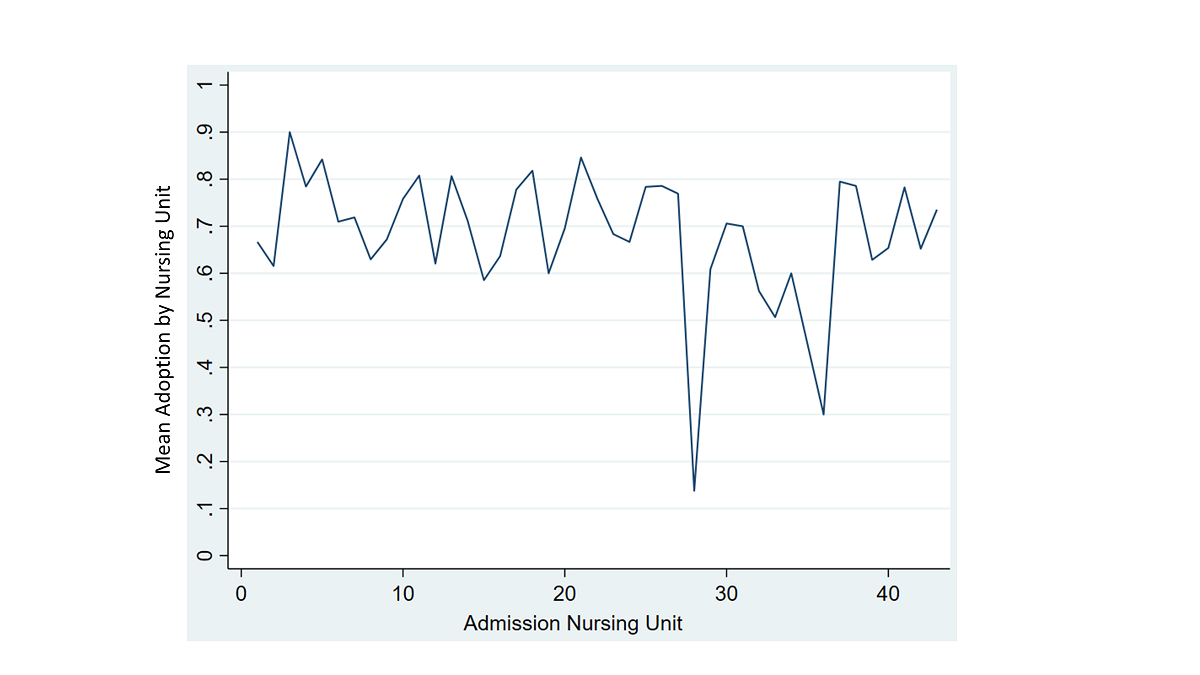

Supplement: Multimedia Appendix 14 [file medinform_v9i11e30743_app14.png]

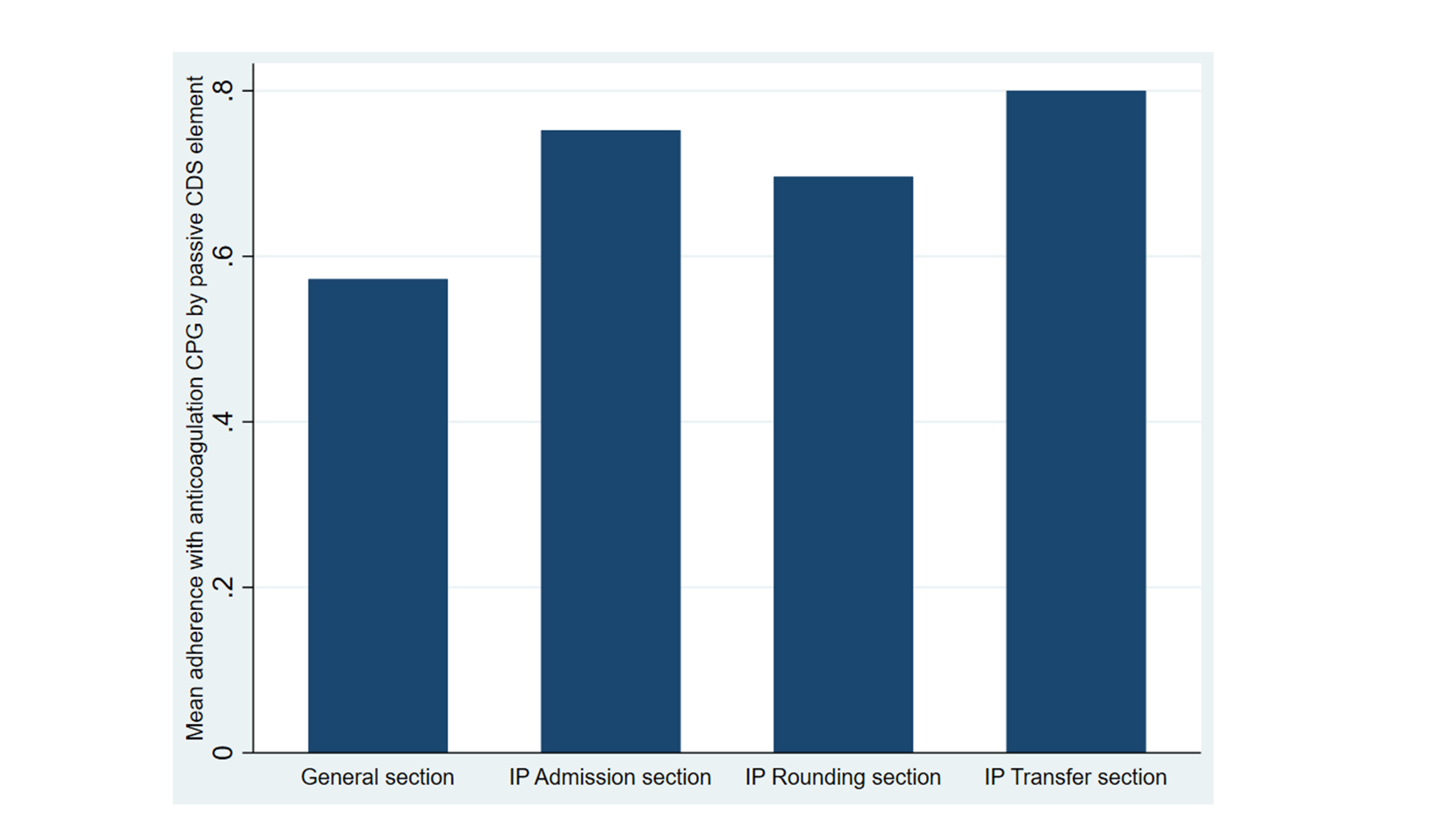

Supplement: Multimedia Appendix 15 [file medinform_v9i11e30743_app15.png]

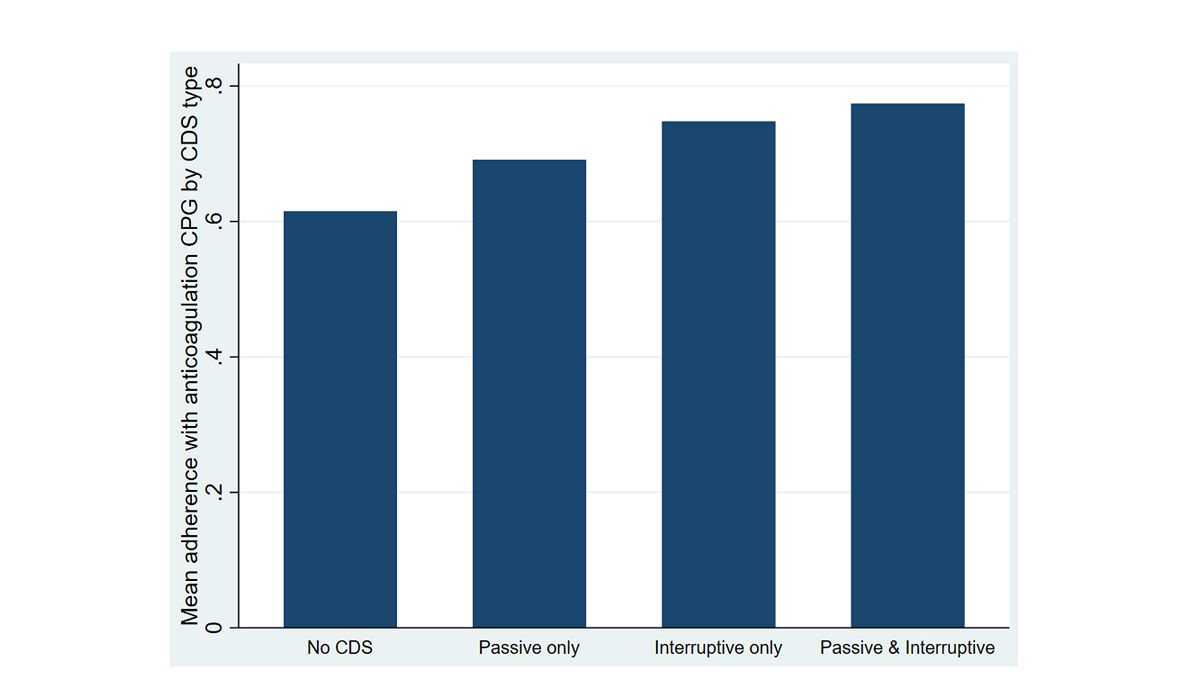

Supplement: Multimedia Appendix 16 [file medinform_v9i11e30743_app16.png]
