# Supplementary material for: Implementation of an Anticoagulation Practice Guideline for COVID-19 via a Clinical Decision Support System in a Large Academic Health System and Its Evaluation: Observational Study
Source: JMIR Med Inform. 2021 Nov 18;9(11):e30743. doi: 10.2196/30743 (PMC8604256; doi:10.2196/30743)
Supplement: Multimedia Appendix 5 [file medinform_v9i11e30743_app5.docx]

**Multimedia Appendix 5.** Propensity score standardized differences prior to and after matching.

|  | Standardized difference (SD) prior to matching (n = 2503) | SD after matching (n = 1,342 [671 patients/cohort]) |
| --- | --- | --- |
| Admission Month | 0.025 | 0.034 |
| Age (years) | 0.338 | -0.069 |
| Elixhauser Comorbidity Index | 0.051 | -0.047 |
| Area Deprivation Index | -0.029 | -0.011 |
| Race | -0.007 | 0.037 |
| Non-English Speaking | 0.026 | 0.023 |
| Gender | 0.153 | -0.03 |
| Received remdesivir | 0.561 | 0.003 |
| Received tocilizumab | 0.11 | 0.008 |
| Received steroid | 0.366 | 0.039 |
| Minimum SBP within first 24 hours | 0.108 | 0.011 |
| Maximum RR within first 24 hours | 0.171 | -0.028 |
| Minimum S/F ratio within first 24 hours | -0.236 | 0.065 |
| Admitting Hospital | -0.092 | 0.012 |
| Body Mass Index | 0.215 | -0.027 |
| Source of Admission | 0.028 | 0.005 |

Legend**:** Standardized differences before and after nearest neighbor propensity score matching

Abbreviations**:** SD, standardized difference; SBP, systolic blood pressure; RR, respiratory rate; S/F, Oxygen saturation to FiO2 ratio
