# Supplementary material for: Implementation of an Anticoagulation Practice Guideline for COVID-19 via a Clinical Decision Support System in a Large Academic Health System and Its Evaluation: Observational Study
Source: JMIR Med Inform. 2021 Nov 18;9(11):e30743. doi: 10.2196/30743 (PMC8604256; doi:10.2196/30743)
Supplement: Multimedia Appendix 8 [file medinform_v9i11e30743_app8.docx]

**Multimedia Appendix 8.** Clinical outcomes.

|  | Did not receive adherence anticoagulation | Received adherence anticoagulation | p-value |
| --- | --- | --- | --- |
| N | 835 | 1650 |  |
| ICU Admission within 48 hours, n(%) | 245 (28.7%) | 443 (26.8%) | 0.32 |
| ICU Admission, n(%) | 262 (30.7%) | 550 (33.3%) | 0.18 |
| Required Mechanical Ventilation, n(%) | 73 ( 8.6%) | 235 (14.2%) | <0.001 |
| All-Cause In-Hospital Mortality, n(%) | 92 (10.8%) | 170 (10.3%) | 0.71 |
| Hospital Length of Stay, median(IQR) | 4.4 (2.1-8.6) | 6.5 (3.9-12.1) | <0.001 |
| Composite Outcome, n(%) | 410 (48.1%) | 933 (56.5%) | <0.001 |
|  |  |  |  |
| VTE Complication, n(%) | 103 (12.1%) | 204 (12.4%) | 0.83 |
| Bleeding Complication, n(%) | 26 ( 3.0%) | 24 ( 1.5%) | 0.007 |
