# Supplementary material for: Implementation of an Anticoagulation Practice Guideline for COVID-19 via a Clinical Decision Support System in a Large Academic Health System and Its Evaluation: Observational Study
Source: JMIR Med Inform. 2021 Nov 18;9(11):e30743. doi: 10.2196/30743 (PMC8604256; doi:10.2196/30743)
Supplement: Multimedia Appendix 9 [file medinform_v9i11e30743_app9.docx]

**Multimedia Appendix 9.** Full model output for multivariable logistic regression evaluating the association of adherence with the clinical practice guideline on hospital admission with the need for intensive care within 48 hours.

|  | OR | 95% CI | P value |
| --- | --- | --- | --- |
| CPG Adherence | **0.39** | **0.30 - 0.51** | **<0.001** |
| Age | 0.99 | 0.98-0.995 | <0.001 |
| Race |  |  |  |
| White | (Ref) |  |  |
| Black | 0.78 | 0.49-1.22 | 0.28 |
| Asian | 0.95 | 0.59-1.51 | 0.82 |
| Hispanic | 1.11 | 0.64-1.89 | 0.72 |
| Declined | 0.70 | 0.42-1.15 | 0.17 |
| Other | 0.90 | 0.37-2.15 | 0.81 |
| Male | 1.37 | 1.07-1.73 | 0.01 |
| ADI quintile |  |  |  |
| 0-19% | (Ref) |  |  |
| 20-39% | 1.28 | 0.87-1.84 | 0.20 |
| 40%-59% | 1.05 | 0.72-1.52 | 0.80 |
| 60%-79% | 1.41 | 0.89-2.22 | 0.14 |
| 80%-100% | 1.08 | 0.63-1.82 | 0.78 |
| Non-English Speaking | 1.32 | 0.92-1.89 | 0.13 |
| Elixhauser Comorbidity Index | 1.05 | 1.01-1.08 | 0.01 |
| BMI | 1.00 | 0.98-1.01 | 0.94 |
| Lowest SBP in first 24 hours (mmHg) | 0.99 | 0.98-0.99 | 0.04 |
| Highest RR in first 24 hours (bpm) | 1.03 | 1.02-1.04 | <0.001 |
| Lowest S/F ratio in first 24 hours | 0.99 | 0.990-0.993 | <0.001 |
| Received Remdesivir | 1.98 | 1.45-2.68 | <0.001 |
| Received Tocilizumab | 4.34 | 2.44-7.71 | <0.001 |
| Received Steroids | 2.24 | 1.65-3.02 | <0.001 |
| Admission Month |  |  |  |
| March | (ref) |  |  |
| April | 0.44 | 0.17-1.103 | 0.08 |
| May | 0.43 | 0.17-1.06 | 0.07 |
| June | 0.37 | 0.14-0.98 | 0.05 |
| July | 0.32 | 0.12-0.83 | 0.02 |
| August | 0.24 | 0.094-0.61 | 0.00 |
| September | 0.27 | 0.10-0.71 | 0.01 |
| October | 0.13 | 0.05-0.33 | <0.001 |
| November | 0.06 | 0.02-0.15 | <0.001 |
| Admission Hospital |  |  |  |
| Hospital 0 | (ref) |  |  |
| Hospital 1 | 1.08 | 0.25-4.60 | 0.92 |
| Hospital 2 | 1.51 | 0.47-4.77 | 0.48 |
| Hospital 3 | 6.25 | 1.64-23.69 | 0.01 |
| Hospital 4 | 1.74 | 0.50-5.98 | 0.38 |
| Hospital 5 | 0.54 | 0.18-1.57 | 0.26 |
| Hospital 6 | 0.32 | 0.10-0.92 | 0.04 |
| Hospital 7 | 0.81 | 0.25-2.58 | 0.73 |
| Hospital 8 | 0.13 | 0.03-0.53 | 0.01 |
| Hospital 9 | 0.43 | 0.14-1.24 | 0.12 |
| Hospital 10 | 1.62 | 0.49-5.28 | 0.43 |
| Hospital 11 | 1.51 | 0.47-4.74 | 0.48 |
| Source of Admission |  |  |  |
| Home | (ref) |  |  |
| ED | 2.26 | 1.08-4.73 | 0.03 |
| SNF | 1.27 | 0.53-2.99 | 0.59 |
| External Hospital Transfer | 4.40 | 1.69-11.36 | <0.001 |
| Admission for Surgery | 2.29 | 0.33-15.68 | 0.40 |
| Clinic | 0.44 | 0.07-2.50 | 0.35 |
